# Physical, functional and sensory properties of bitter chocolates with incorporation of high nutritional value flours

**DOI:** 10.3389/fnut.2022.990887

**Published:** 2022-09-20

**Authors:** Luz Quispe-Sanchez, Marilu Mestanza, Malluri Goñas, Elizabeth Renee Ambler Gill, Manuel Oliva-Cruz, Segundo G. Chavez

**Affiliations:** ^1^Instituto de Investigación para el Desarrollo Sustentable de Ceja de Selva, Universidad Nacional Toribio Rodríguez de Mendoza de Amazonas, Chachapoyas, Peru; ^2^College of Life Sciences and Agriculture COLSA, University of New Hampshire, Durham, NC, United States

**Keywords:** cañihua, kiwicha, calorimetry, rheology, antioxidant activity

## Abstract

Due to the growing demand for healthy food products, the industry is seeking to incorporate inputs with high nutritional potential to traditional products. The objective of this research was to evaluate the effect of incorporating *Lepidium meyenii*, *Chenopodium pallidicaule*, *Amaranthus caudatus*, *Sesamum indicum* and *Salvia hispanica* flours on the physical, chemical, rheological, textural and thermal characteristics, and the degree of sensory acceptance of dark chocolate bars (65% cocoa). To this end, chocolate bars were made with the incorporation of five flours in four doses (1, 2, 3 and 4%), obtaining 20 different formulations compared with a control treatment (without flour addition). It was found that as flour incorporation levels increased, viscosity, antioxidants and particle size of the chocolates increased, but hardness and pH decreased. The addition of the flours also affected the acceptability and microstructure of the chocolate bars. The incorporation of up to 4% of the flours studied improved the degree of acceptance of the chocolates. Consequently, the incorporation of grain flours with high nutritional value can enhance the characteristics of dark chocolates, becoming a technological alternative for the chocolate industry.

## Introduction

Among the most popular foods consumed by people of all ages is chocolate. Their consumption is associated with beneficial antioxidant properties that reduce oxidative stress, help prevent cardiovascular disease and improve overall health (1–3). On the other hand, chocolate consumption can improve mood states and cognitive and sensory responses (4); This sensory theory is related to the melting profile in the mouth, and chocolate’s aromatic characteristics that are impacted by the cultivation, harvesting, post-harvesting of cocoa, and processing to obtain chocolate (5).

Global chocolate consumption has been increasing, and the trend toward dark chocolate consumption by individuals has been increasing, as it is considered a healthy, sustainable and high-quality alternative. For example, European countries such as Italy prefer dark chocolates by 46%, followed by chocolate with hazelnut (23%) and chocolates with different additional ingredients (16%) (6).

The EU directive 2000/36/EC of the European Parliament of the European Union has simplified legislation on the use of new ingredients in the manufacture of chocolates, which has opened up the possibility for chocolate manufacturers to develop promising new products. Thus, it is possible to improve not only the nutritional functionality of chocolates and cocoa derivatives, but also to reduce the proportion of cocoa or expensive inputs by substituting them with soluble and insoluble fibers, vitamins, minerals, herbal extracts or other inputs (7). However, in chocolates, the processes of incorporation and/or modification, due to their fatty nature (8), could generate considerable physicochemical changes that modify their sensory properties, affecting consumer perception.

On the other hand, the food industry has implemented rigorous innovation strategies and improved product development as competitive tools (9, 10). This is why considerable changes are being experienced in the cocoa and chocolate industry in response to new consumption trend (11). Such is the case in the United States of America, the consumption of chocolates as functional foods has gained popularity as chocolate is consumed to relax, boost the immune system, and even to improve mood (12, 13).

During the last few years, the trend to consume healthy and safe foods has increased greatly (14). Consumers, in addition to demanding safety and nutrition, seek foods that benefit their health and help prevent diseases (15). In addition to price and preferences, another factor driving food purchases is the food additives incorporated at the time of processing (16, 17). These factors cause manufacturers to take consumer preferences into account when developing and launching new products on the market.

Therefore, the incorporation of new inputs with high nutritional potential can be a good alternative. Maca (Lepidium meyenii), popularly known for improving reproductive health, is a good alternative (18), also acts as an anti-inflammatory (19), anti-fatigue (20), antioxidant (21) and as a stimulant to improve learning and memory processes (22, 23). Cañihua (*Chenopodium pallidicaule*), due to its high flavonoid content acts as a potent antioxidant (24, 25). Kiwicha (*Amaranthus caudatus*), has antidiabetic and antihypercholesterolemic effects (26, 27); therefore, it is used as a primary precursor of drugs and food supplements (28–30). Also, pharmacological activities of ajonlojí (*Sesamum indicum*) have been reported as anticancer, antipyretic, antihypertensive, hepatoprotective and antirheumatoid (31, 32). On the other hand, several researches have focused on studying the properties and applications of chia mucilage (*Salvia hispanica*) for its high capacity to retain water and modify the texture of foods (33, 34), in addition, due to its high dietary fiber content, it could act as a supplement for the treatment of obesity (35). These foods whose flours are characterized by their high nutritional value (25, 36) could be incorporated into chocolate formulations.

However, aspects such as particle size and flour composition could have a direct impact on the physical, chemical, rheological and even sensory properties of the food (37–39). Además, the refining process to which cocoa nibs are subjected to obtain chocolate could positively affect the availability of nutrients from the meals, as it involves prolonged cell breakdown (37, 40) and they could be encapsulated by the fatty matrix of the product.

To guarantee the quality of chocolates during the production process, aspects such as rheology, texture, physical and chemical properties, which influence sensory characteristics, are monitored (41). In addition, the objective of this research was to study the effect of the incorporation of high nutritional value flours on the physical, functional and sensory properties of dark chocolate.

## Methodology

### Cocoa and cereals production

The fermented and dried cocoa beans, variety CCN-51 (7.5% moisture), were provided by the Research Institute for Sustainable Development of Ceja de Selva of the UNTRM, obtained from cocoa plantations in the district of Cajaruro, province of Utcubamba, Amazonas region, Peru.

**FIGURE 1:**
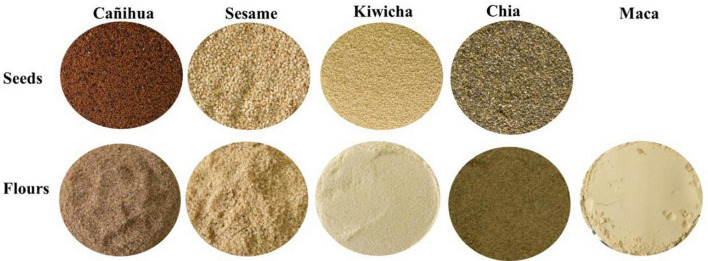
Seeds and flours used in the experiment.

The grains (sesame, cañihua, kiwicha and chia) were purchased at the Central Market in the city of Chachapoyas. In the Cocoa Quality Control Laboratory, here they were removed of impurities, washed and de-saponified with water (kiwicha beans) for 15 min at 75°C. They were then roasted at 110°C for 30 min and ground in a disk mill. The flours obtained were sieved on 850 μm sieve to homogenize. n the case of maca flour, it was purchased, processed and packaged at the same place. Figure 1 shows the seeds and flours used in the experiment, where the cañihua and chia flours had a dark brown color and the maca, kiwuicha and sesame flours had a cream color.

### Cocoa paste

Cocoa beans were roasted in a forced convection oven (Venticell Ecoline, Germany) at a temperature of 120°C for 40 min. They were then dehulled in an industrial mill (A&Z, Peru). Once dehulled, the nibs were crushed in a disk mill and refined for 10 h in 3 kg capacity conchers (Premier, India).

### Experimental procedure

A 5Ax4B bifactorial experiment was carried out, where A was the type of flour (maca, sesame, cañihua, kiwicha and chia) and B the incorporation dose (1, 2, 3 and 4%). As a result, there were 20 treatments (formulations) and a control treatment (chocolate without flour incorporation). After 10 h of conching-refining of the cocoa nibs, the flours were added, trying to obtain chocolates with 65% cocoa. The refining process continued for 11 more hours. Subsequently, tempering was carried out by raising the temperature to 48°C, then lowering it to 28°C and raising it to 32°C. Bars of 50 g (12 × 5 × 0.5 cm^3^) and lozenges of 0.75 g (1 × 1 × 0.5 cm^3^) were molded. The chocolates (tablets) were wrapped in aluminum foil, packed in hermetically sealed PET bags and stored under refrigeration (4–8°C). All treatments were performed in triplicate.

### pH

The pH was determined by the AOAC 981.12/90 method (42) with a digital potentiometer (HANNA instruments).

### Particle size

Particle size was determined following the procedure described by Ibrahim et al. (43) with some modifications. A solution of melted chocolate in 50% sunflower oil was prepared in a beaker. A drop of the solution was added inside the jaws of the micrometer (Mitutoyo-2017) and closed for reading.

### Microstructure

For microstructure analysis, an inverted fluorescence microscope (IX83, Olympus, Tokyo, Japan) with a 40× objective with high resolution polarized light, equipped with a camera (Nikon D810, Tokyo, Japan), was used. For this purpose, melted chocolate at 55°C was placed on a glass slide (44) and micrographs were obtained to observe the distribution and size of the particles in the chocolate.

### Hardness

A CTX texture analyzer (AMETEK Brookfield) with TexturePro 1.0.19 software was used, equipped with a 10 kg load cell. The test was performed with a 30°conical probe, a test speed of 1 mm/s and a depth of 0.8 mm. The pre-test speed was 5 mm/s, the post-test speed was 10 mm/s, the data acquisition rate was 10 points per second, and the activation force was 5 g. The hardness in grams of the tablets of the different chocolate formulations was obtained.

### Rheological properties

Rheological properties were measured using a modular compact rheometer (Anton Paar, model MCR 302e, Austria), equipped with a CC27 concentric cylinder geometry installed in a Peltier system. The chocolates were pre-melted at 40°C in an oven (Venticell Ecoline, Germany) for 60 min. Rheological measurements were then carried out at 40°C. The measurement cycle started with a preconditioning at 40°C for 60 s, then a shear rate of 5 s-1 was applied for 100 s. Subsequently, the shear rate was increased from 2 to 50 s-1. The data were processed by the equipment software (RheoCompass vs s 1.30) following the Casson model (Equation 1), where σ (Pa) is the shear stress, σ0 is the Casson yield stress (Pa), K1 is the consistency index (Pa. s) and γ (s-1) is the shear rate [Glicerina et al. (45)] and numeral 209 of the International Confectionery Association technical standard (46).


(1) $$\sigma^{0.5}={\left(\sigma_{0}\right)}^{0.5}+K_{1}\,{\left(\gamma \right)}^{0.5}$$

### Calorimetric properties

The thermal behavior of chocolates formulated with cereals was measured based on the method of Toker et al. (47), by differential scanning calorimetry (DSC-60 plus, Japan). For this purpose, samples were weighed on an analytical balance (2.5 ± 0.5 mg) and placed in aluminum vials, sealed tightly and subjected to the oven. An empty, sealed aluminum cuvette was used as a blank. Nitrogen gas (25 ml/min) at normal pressure was used. The samples were subjected to a temperature ramp from 0 to 60°C for 20 min to melt all crystals. All measurements were performed in triplicate.

### Sensory evaluation

The degree of acceptance of the chocolates obtained with each treatment was evaluated following the procedure of the Spanish Standard UNE-ISO 8587 (48) by 24 untrained judges at the Cocoa and Coffee Quality Control Laboratory. A balanced incomplete block design was employed in two series of seven random distributions taking into account the conditions established in the Standard.

### Antioxidant activity

Samples were defatted following the procedure described by Suazo et al. (49). For this purpose, 500 mg of chocolate were weighed and placed in a 15 mL falcon tube, mixed with 3 mL of hexane and shaken at 3,000 rpm for 10 min. The mixture was then centrifuged at 4,830 rpm for 20 min in a centrifuge (PrO-Analytical, Britain) at room temperature. The process was repeated 4 times, then the supernatant was removed and the defatted chocolate powder was recovered. The defatted powders were left for 24 h in a fume hood to remove the solvent in the dark.

Next, methanolic extracts were obtained using the method described by Jonfia-Essien, West, Alderson and Tucker (50) with some modifications. For this, 0.1 g of defatted sample was taken, mixed with 10 ml of 80% methanol solution, shaken for 15 min in a magnetic stirrer (Velp. Scientifica) at room temperature. Then, it was centrifuged at 4,830 rpm for 30 min, filtered on filter paper (Whatman N° 40–2.5 μm) and the supernatant was stored in amber bottles with lids at −24°C until further analysis.

### DPPH

DPPH analysis (% free radical uptake) was performed following the method reported by Brand-Williams et al. (51), which indicates that DPPH must first be prepared in 20 mg/L methanol. Then, a methanolic solution of the sample (Solution A1) to be analyzed was prepared at a concentration of 300 μg/ml, from which 0.75 ml was taken to prepare the sample blank + 1.5 ml of methanol (A3) and 0.75 ml to prepare the sample + 1.5 ml of DPPH methanolic solution (A2), left for 5 min in the dark and the absorbance was read at 517 nm in the spectrophotometer (T 9200 PEAK Instruments, United States). Finally, with the values of the absorbances obtained, the DPPH was determined according to the formula of Equation 2, where A0: Absorbance of the DPPH solution, AS: Absorbance of methanol, AT: Absorbance of the sample.


(2) $$\%inhibitionofDPPH=\frac{\left(A\,0-A\,S\right)-\left(A\,T-A\,S\right)}{\left(A\,0-A\,S\right)}X100$$

## Results and discussion

### Particle size, sensory acceptability, pH, hardness and antioxidant activity

The particle size of the chocolate bars is different according to the type of flour incorporated into the formulation (Table 1). The higher degrees of flour incorporation (3 and 4%) allowed obtaining chocolates with lower hardness (from 6,716 to 3,677 g), agreeing with previous works that showed that the inclusion of other foods in the formulations affects the rheological and visual properties of the chocolates (52).

**TABLE 1:** Results of particle size, sensory acceptance, hydrogen potential, hardness and antioxidant activity of dark chocolates enriched with 5 types of high-nutritional value flours, in different percentages.

Types of flours	P (%)	Particle size (μm)	Sensory acceptance	pH	Hardness (g)	Antioxidant activity (mmol TE/L)
Maca	1	56.7 ± 3.1cd	3.41 ± 0.72a	5.62 ± 0.01cd	6,716 ± 63.7 a	90.2 ± 0.20f
	2	26.7 ± 3.2ef	3.04 ± 0.75ab	5.21 ± 0.01i	5,587 ± 82.7 d	87.9 ± 0.32f
	3	51.3 ± 0.6cde	3.17 ± 0.81b	5.55 ± 0.01de	4,822 ± 19.6 g	100 ± 0.56a-d
	4	144 ± 2.9a	2.54 ± 0.59a	5.48 ± 0.01ef	4,506 ± 57.7 h	100 ± 0.15a-d
	1	27.0 ± 2.7ef	2.67 ± 0.76ab	5.45 ± 0.01b	6,288 ± 70.7 d	82.2 ± 5.02a-c
	2	20.0 ± 2.7f	2.91 ± 0.97a	5.31 ± 0.01h	5,684 ± 15.9 f	99.6 ± 0.90c-e
Sesame	3	43.3 ± 7.5cdef	2.25 ± 0.61b	5.47 ± 0.01cd	5,014 ± 10.0 j	101 ± 0.17a-d
	4	36.0 ± 1.0def	2.42 ± 0.58ab	5.43 ± 0.01hi	4,275 ± 15.3 e	102 ± 0.45a-d
	1	43.7 ± 0.6cdef	3.13 ± 0.94a	5.66 ± 0.08c	6,462 ± 9.9 b	99.9 ± 0.41 g
	2	34.3 ± 3.5cd	2.79 ± 0.83 a	6.34 ± 0.11a	4,372 ± 5.8 ij	100 ± 0.12 b-d
Cañigua	3	56.0 ± 1.0def	2.67 ± 0.76 a	5.61 ± 0.01cd	4,392 ± 5.7 ij	99.9 ± 2.20 ab
	4	33.3 ± 2.9def	2.91 ± 0.71a	5.35 ± 0.02gh	3,677 ± 9.8 l	99.4 ± 0.53 a
	1	39.3 ± 4.8cdef	3.37 ± 0.49a	5.61 ± 0.01cd	5,425 ± 3.2 hi	96.2 ± 1.46a-d
	2	26.0 ± 3.5ef	3.04 ± 0.75ab	5.29 ± 0.02hi	4,503 ± 5.8 j	98.5 ± 0.98a-d
Kiwicha	3	63.0 ± 3.5c	2.50 ± 0.59bc	5.42 ± 0.03fg	4,284 ± 1.5 k	100 ± 0.31 a-d
	4	96.0 ± 2.7b	2.21 ± 1.10c	5.12 ± 0.01j	4,058 ± 55.1 b	100 ± 0.39b-d
	1	33.7 ± 2.9def	3.00 ± 0.66 a	5.79 ± 0.01 f	5,241 ± 5.7 e	100 ± 0.19e
	2	26.0 ± 2.0ef	3.00 ± 0.65 a	5.30 ± 0.01h	4,881 ± 5.8 fg	98.6 ± 1.87d-e
Chia	3	50.3 ± 0.6cde	3.00 ± 0.65 a	5.61 ± 0.01ef	3,780 ± 5.8 l	100 ± 0.30a-c
	4	39.7 ± 6.7cdef	2.58 ± 0.97 a	5.29 ± 0.01fg	2,587 ± 5.2 m	100 ± 0.13 a-d
Control	0	22.7 ± 1.2f	1.95 ± 0.75d	5.69 ± 0.02c	5,613 ± 5.8 d	82.5 ± 0.54 g

*Different letters indicate statistically different groups, according Tukey (*p* < 0.05; *n* = 3).

Similarly, the pH of the chocolates was slightly modified for all treatments, decreasing to 5.21 for sesame flour; which may be due to the chemical composition of the flour (starch, fatty acids) (53).

The dark chocolate formulations with the addition of flours had particle sizes between 20 and 144 μm, while the control chocolate (without flour) had a particle size of 22.7 μm. Sesame, cañigua and chia flours had the least effect on the particle size of the chocolate (< 56 μm). In contrast, when kiwicha and maca flours were incorporated, the particle size increased considerably, up to 96 and 144 μm, respectively (Table 1). Article size in chocolates is very important in sensory perception; in fact, a chocolate could be perceived as gritty when the particles are larger than 25 μm (44). Table 1 also shows that the incorporation of flours improves the degree of sensory acceptability of dark chocolates, since all treatments scored higher than the control treatment (*p* < 0.05), highlighting the treatments with incorporation of cañigua and chia flour in all degrees of incorporation, which reached acceptability scores between 2.58 and 3.13. Previous studies have already demonstrated that chocolate flavor is influenced by the composition of the added inputs (54), this theory can support the different degrees of acceptability of the chocolates obtained in this research.

The incorporation of the flours increased the antioxidant capacity of the chocolates. With the addition of the flours, chocolates with higher antioxidative activity were obtained compared to the control treatment which had 82.5 mmol/L TE (Table 2). Evidently, there is a direct effect on the content of antioxidant compounds by the addition of the flours. In addition, the incorporated antioxidant compounds can improve the nutritional and funtional value, the chemical contribution and can stabilize the oxidation of chocolate fats during storage (55).

**TABLE 2:** Rheological properties of dark chocolates enriched with high nutritional value flours.

Type of flour	P (%)	Casson plastic viscosity (Pa. s)	Casson yield stress (Pa)	*R* ^2^
	1	1.922 ± 0.009i	21.942 ± 1.589f	0.999
	2	1.935 ± 0.005í	18.627 ± 0.059g	0.999
Maca	3	2.945 ± 0.007d	18.758 ± 0.000g	0.999
	4	3.972 ± 0.000a	26.176 ± 0.903e	0.999
	1	2.266 ± 0.073fg	35.451 ± 0.490cd	0.999
	2	2.761 ± 0.119e	35.451 ± 0.110bc	0.999
Ajonjolí	3	2.879 ± 0.066de	35.612 ± 0.578bc	0.999
	4	2.931 ± 0.026d	37.563 ± 0.083a	0.095
	1	1.518 ± 0.043j	18.427 ± 0.629g	0.999
	2	2.364 ± 0.015f	21.805 ± 0.540f	0.999
Kiwicha	3	2.828 ± 0.058de	34.181 ± 0.000cd	0.999
	4	2.867 ± 0.020de	35.557 ± 0.126bc	0.999
	1	2.159 ± 0.004gh	26.747 ± 1.348e	0.999
	2	2.196 ± 0.008fgh	25.565 ± 0.880e	0.999
Cañihua	3	2.304 ± 0.036fg	37.538 ± 0.178a	0.999
	4	3.463 ± 0.125b	38.354 ± 0.084a	0.999
	1	2.069 ± 0.017hi	27.378 ± 0.309e	0.998
	2	3.126 ± 0.023c	33.343 ± 0.508d	0.998
Chía	3	3.140 ± 0.038c	37.184 ± 0.219ab	0.999
	4	3.567 ± 0.112b	38.852 ± 0.158a	0.998
Control	0	1.588 ± 0.009i	15.300 ± 0.404h	0.999

*Different letters indicate statistically different groups, according Tukey (sig. = 0.05; *n* = 3).

On the other hand, darker flours (cañihua and chia) increased the antioxidant capacity of chocolates to a greater extent than lighter flours (maca and sesame), which may be due to the fact that foods with more color contain more phenolic compounds and greater antioxidative activity (56).

Therefore, the chemical composition of flours can influence the physical, chemical and sensory properties of chocolates. For example, the inclusion of lentil flour has been studied and depending on the matrix in which it is incorporated, it modifies the rheological, textural and sensory acceptance properties of the final product (57, 58).

As shown in Table 1, all flours modified the characteristics of the chocolates with respect to the control. Flour additions increased particle size, but all treatments significantly improved (*p* < 0.05) the degree of sensory acceptance, increased pH and antioxidant activity. On the other hand, doses higher than 2% reduced the hardness of the chocolate bars. Consequently, based on the degree of acceptance and antioxidant activity with 0.05 of statistical significance, the best alternatives would be: maca 4%, sesame 2%, cañigua 1–4%, kiwicha 1% and chia 1–4%.

### Microstructure

**FIGURE 2:**
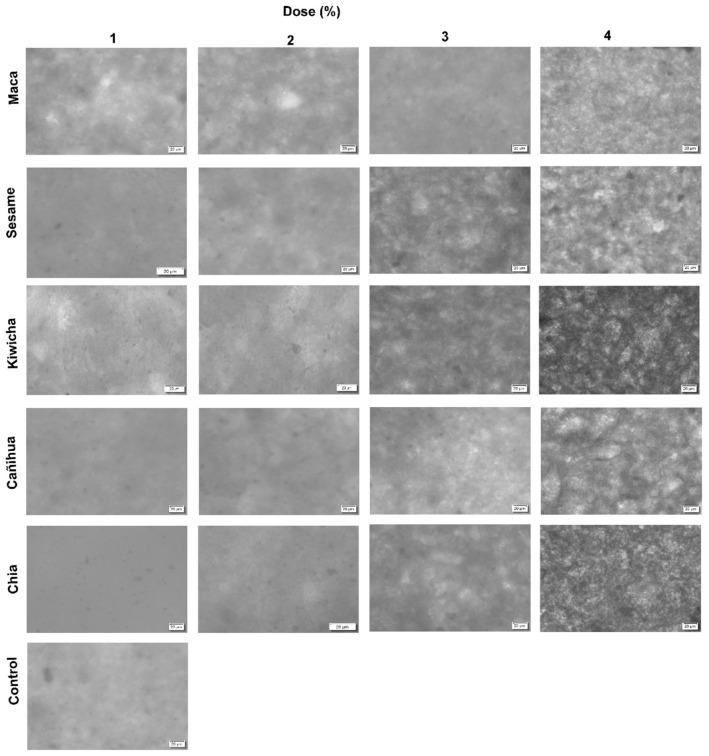
Micrograph of dark chocolates enriched with high-nutritional value flours in different percentages.

As the flour content increases, the microstructure of the chocolates is affected (Figure 2). The incorporation of up to 2% of chia and cañigua flours makes it possible to obtain chocolates with a more uniform structure; however, levels higher than 2% increase the granulometry of the final product. Por tanto, kiwicha, sesame and maca flours affect the microstructural aspect of the chocolates. Previous studies showed that the incorporation of flours affects the microstructure of chocolates (8), interfering in the fat classification and crystallization process (59).

The effects found in this study are variable due to the nature of each source. For example, chia flour, in addition to containing fats, is an important source of gummy elements (60) that can help in the structure of chocolate bars. As it was evidenced that the addition of chia flour in bread increases the moisture and lipid content (61) because these elements represent more than 30% of its composition (62, 63).

### Textural profile of chocolates with flours

**FIGURE 3:**
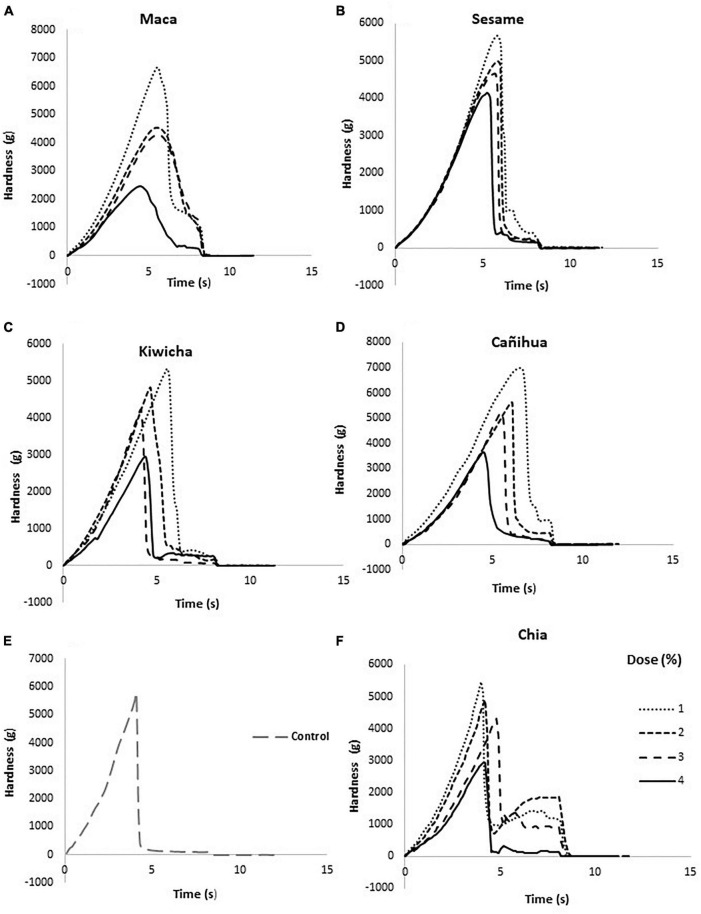
Typical texture curves of dark chocolates with the addition of flours. **(A)** Maca, **(B)** ajonjolí, **(C)** kiwicha, **(D)** cañihua, **(E)** chia, and **(F)** with no flours.

Table 1 shows that the addition of different types of flour in the formulations also modifies the hardness of the chocolates (from 2,587 to 6,716 g). This has already been demonstrated in previous studies (64). However, high levels of flour (more than 3%) reduce the hardness of chocolates, which could be due to the fact that flours modify the viscosity of the paste (during the refining process) and interfere in the fat sorting and crystallization process (59). Texture profiles are presented in Figure 3, where chocolate bars with higher contents of maca, sesame, cañigua, kiwicha, and chia flour took less time to fracture (5 min approx.) and unlike the other flours, they offered resistance until the course was completed (Figure 3E), which illustrates the action of the flours of these seeds on the physical property of the food (65, 66).

### Rheological properties

As previous studies have shown, the rheological properties of chocolate are affected by the incorporation of other products and, particle size is inversely correlated with the firmness, consistency, cohesiveness, viscosity and hardness of chocolate bars (67, 73). Smaller particles have also been found to favor higher dispersibility of chocolate ingredients, because they occupy the spaces between larger particles, leading to a lubricating effect with a consequent reduction in viscosity (68). Tighter results could be obtained by conditioning other process factors such as refining, time, process temperature and tempering of the chocolate bars.

In this study, the rheological properties of chocolates with incorporated cereal flour were studied at steady state and plastic viscosity. Dark chocolates have a complex rheological behavior (non-Newtonian liquid) showing an apparent yield strength and plastic viscosity that depends on the manufacturing process (69). The incorporated flours modified the rheological properties of the chocolate bars to different degrees; in fact, with 4% incorporation of maca, cañihua and chia, chocolates with viscosities higher than 3.5 Pa.s were obtained, and with sesame and kiwicha, plastic viscosities lower than 2.93 Pa.s were obtained (Table 2).

The determination of the rheological properties of chocolate is crucial to develop high quality products with a well-defined texture (70). As shown in Table 2, the incorporation of flours increases the viscosity of the chocolates, with cañihua and chia flours having the greatest effect on this property. Bourré et al. (71) demonstrated that there is greater starch damage with intense flour milling (< 500 μm), increasing water absorption capacity and viscosity.

### Calorimetric properties

**FIGURE 4:**
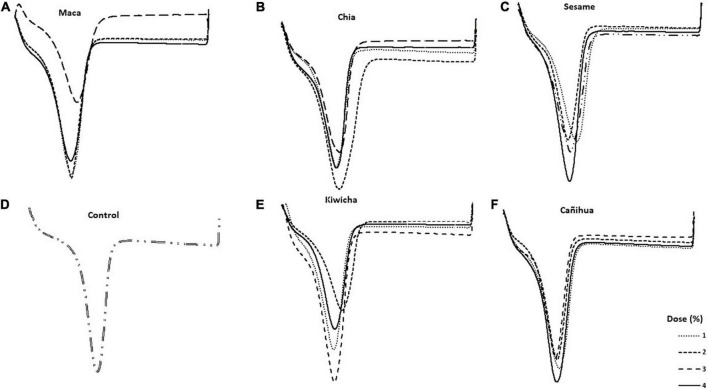
Typical differential scanning calorimetry curves of dark chocolates with the addition of flours. **(A)** Maca, **(B)** ajonjolí, **(C)** kiwicha, **(D)** cañihua, **(E)** chia, and **(F)** with no flours.

Figure 4 shows the thermograms obtained for each treatment. Although the enthalpy varies according to the type and percentage of flour added, we observed that when the amount of flour in the formulations is increased, the melting temperature of the chocolate also increases. The values found ranged from 31 to 37°C for all treatments, which is close to the optimal range (31.5–32°C) described by other authors (8). Indeed, tempering conditions are fundamental and should be taken into account in future studies, addressing tempering models (72).

## Conclusion

The results of this study revealed that the incorporation of flours has considerable potential as an alternative to synthetic additives in chocolates by improving their antioxidant capacity. Indeed, the incorporation of flours significantly increased the antioxidant activity of the chocolate samples; however, there was a slight difference in the rheological and melting properties. The addition of up to 4% of chia and cañihua flours to chocolates resulted in chocolates with higher sensory acceptability than chocolates with sesame, kiwicha, maca and even the control treatment. Our finding will contribute to continue developing new products, improving the rheological and sensory properties of chocolate.

## Data availability statement

The original contributions presented in this study are included in the article/supplementary material, further inquiries can be directed to the corresponding author.

## Author contributions

MO-C and SC: conceptualization. LQ-S and MM: methodology. SC, LQ-S, and MM: formal analysis. MO-C, ERAG, and MG: research and writing—preparing the original draft. LQ-S, MM, MG, ERAG, and SC: writing—revision and editing. MO-C, ERAG, SC, and MG: funding acquisition. All authors contributed to the article and approved the submitted version.
