# Correction: Physical, functional and sensory properties of bitter chocolates with incorporation of high nutritional value flours

**DOI:** 10.3389/fnut.2026.1799462

**Published:** 2026-03-13

**Authors:** Luz Quispe-Sanchez, Marilu Mestanza, Malluri Goñas, Elizabeth Renee Ambler Gill, Manuel Oliva-Cruz, Segundo G. Chavez

**Affiliations:** 1 Instituto de Investigación para el Desarrollo Sustentable de Ceja de Selva, Universidad Nacional Toribio Rodríguez de Mendoza de Amazonas, Chachapoyas, Peru; 2 College of Life Sciences and Agriculture COLSA, University of New Hampshire, Durham, NC, United States

**Keywords:** cañihua, kiwicha, calorimetry, rheology, antioxidant activity

There was a mistake in Figure 2 as published. The original figure contained an incorrect version due to an error during figure preparation. Column 1 and 2 of Sesame were identical and column 2 and 3 of Kiwicha were also identical.

The corrected Figure 2 appears below.

**Figure 2:**
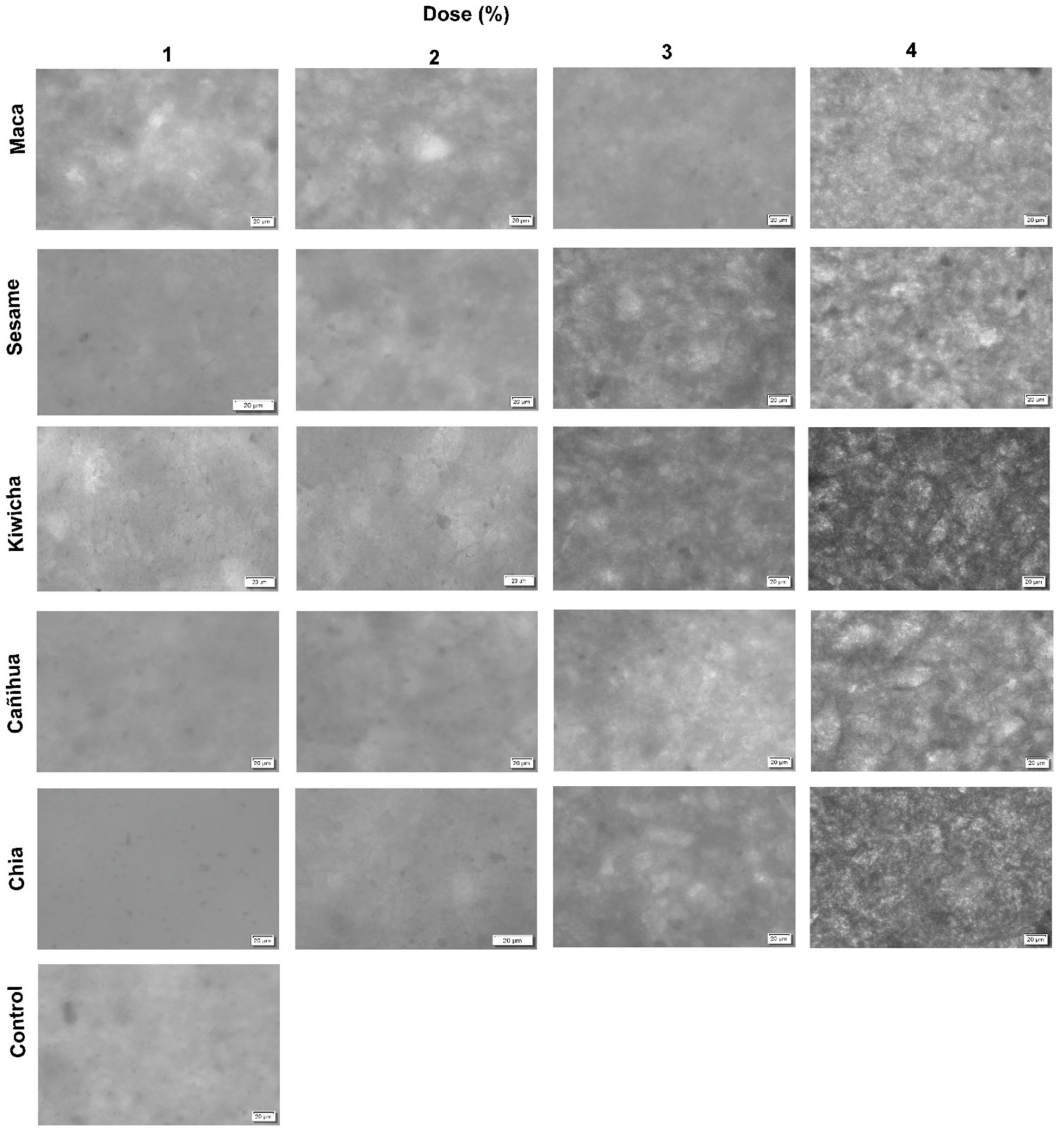
Micrograph of dark chocolates enriched with high-nutritional value flours in different percentages.

The original version of this article has been updated.

